# Identifying Quantum Interference Effects from Joint Conductance–Thermopower Statistics

**DOI:** 10.1021/acs.nanolett.4c04439

**Published:** 2024-11-13

**Authors:** Justin P. Bergfield

**Affiliations:** † Department of Physics, Illinois State University, Normal, Illinois 61790, United States; ‡ Department of Chemistry, Illinois State University, Normal, Illinois 61790, United States

**Keywords:** quantum interference, quantum transport, molecular thermopower, molecular conductance, many-body theory

## Abstract

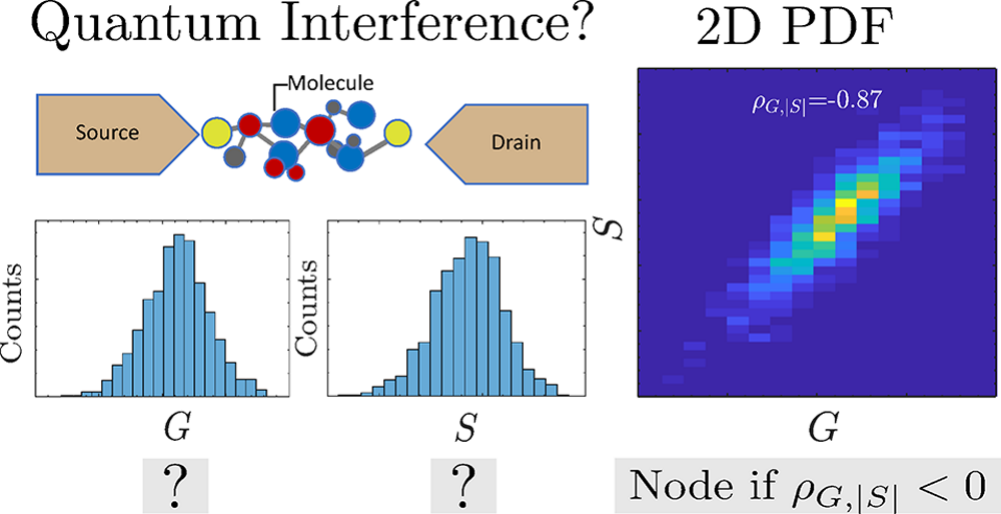

Although quantum effects are thought to dominate the heat and charge transport through molecular junctions, large uncertainties in chemical structure, lead-molecule coupling strengths, and energy levels make it difficult to definitively identify these effects from the measured thermopower *S* and conductance *G* distributions alone. Here, we develop a simple statistical method to identify destructive quantum interference features (nodes) through the anticorrelation between simultaneously measured *G* and *S* values. We find these correlations can be used to unambiguously identify far-detuned nodes, even when *G* and *S* distributions alone cannot. As an example, we consider several para- and meta-configured systems, including benzenediamine and diiodo-terphenyl-based junctions, finding that nodes can be identified in ensembles with broad level-alignment and lead-molecule coupling distributions, and with significant anodal transport contributions, including from vacuum tunneling. The efficacy and limitations of this method are analyzed.

Quantum coherence is a critical resource needed to realize a variety of novel quantum devices, with applications spanning thermal energy conversion,^1−4^ electronics,^5−9^ sensing,^10^ light-harvesting,^11^ and quantum information processing.^12^ In this work, we focus on identifying transmission nodes, destructive interference features in the electronic transport spectrum whose presence requires quantum coherence. These nodes are uniquely quantum in nature, occurring only at energies where all coherent electronic transmission amplitudes cancel completely. While certain nodes can be explained by nanostructure or junction symmetry,^5,6,13−16^ others arise from the structure of the many-body Hilbert space and may not have single-particle or configuration-space explanations.^15,17−19^ As such, nodes are important not only as indicators of quantum-dominated transport but also as probes of fundamental aspects of interacting systems.

Although interference is a general consequence of coherence, we focus on transport through single-molecule junctions (SMJs), systems composed of small organic molecules coupled to macroscopic electrodes, for a few important reasons. The transport through these systems is predominantly quantum coherent and elastic, even at room temperature and in noisy chemical and electromagnetic environments,^20−23^ allowing quantum interference (QI) effects such as nodes to be directly observed in the transport spectra.^24,25^ Additionally, molecular and junction symmetries can be used to control QI,^3,13,14^ allowing exploration of its various aspects. Finally, although incoherent transport channels are always present, rational chemical design reduces their influence.

Given the challenges of precisely reproducing SMJ transport, experimental methods typically rely on repeated measurements to construct characteristic transport *distributions*, even for ensembles with significant variations in level alignment, coupling strength, etc.^26^ These methods have been used to indirectly identify nodes by comparing conductance distributions of nodal and anodal isomers, such as meta- and para-phenyl bridges.^20,21,27^ Direct node observation may also be possible by examining how gating and bias voltages differentially influence isomer distributions.^24,25^ The joint probability distribution , constructed by binning *simultaneously* measured *G* and *S* data,^28−33^ captures not only the average conductance and thermopower but also correlations between them.

In this article, we demonstrate that transmission nodes can be identified through anticorrelations between simultaneously measured *G* and *S* values, quantified by the condition ρ_*G*,|*S*|_ < 0, where ρ is Pearson’s correlation coefficient. Using many-body theory and a reduced-orbital model, we calculate the transport distributions for para- and meta-configured benzenediamine (BDA) and diiod*o*-terphenyl junctions, showing that this correlation condition serves as an unambiguous indicator of a node, directly tied to quantum transport theory, and is effective even in systems with significant uncertainties in energy-level alignments, lead-molecule couplings, and configurations.

This method is also insensitive to the exact energy of the node and remains effective as long as the lead’s chemical potential is closer to the node than to the nearest molecular resonance (e.g., the HOMO or LUMO levels). However, in cases where variations of *G* or *S* over the ensemble due to molecular transport are small relative to factors like statistical noise, the correlation coefficient may approach zero, allowing noise to dominate. Additionally, the correlation signature may be obscured in systems with significant anodal contributions, such as those arising from various incoherent or off-resonant processes. Despite these constraints, we find the correlations between simultaneously measured quantities provides valuable insights into the nature of transport.

In linear-response, the electrical conductance and thermopower may be expressed as
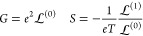
1where  is the *n*th order Onsager function. At room temperature, SMJ transport is primarily coherent and elastic, allowing these to be written as
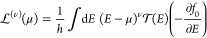
2where *f*_0_(*E*) = [exp((*E* – μ_0_)/*kT*_0_) + 1]^−1^ is the Fermi–Dirac distribution with chemical potential μ_0_ and temperature *T*_0_.

We utilize nonequilibrium Green’s function (NEGF) theory to describe the transport, where the transmission may be expressed in this regime as^34^
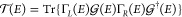
3where  is the junction’s retarded Green’s function. The tunneling-width matrix for contact α may be expressed as
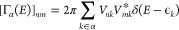
4where *n* and *m* label π-orbitals within the molecule, and *V*_*nk*_ is the coupling matrix element between orbital *n* of the molecule and a single-particle energy eigenstate ϵ_*k*_ in electrode α. We consider transport in the broad-band limit, treating this matrix as energy-independent. In many-body molecular Dyson equation (MDE) theory, the Green’s function of a SMJ is given by^34^
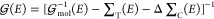
5where  is the molecular Green’s function, Σ_T_ represents the tunneling self-energy matrix with ∑_T_ = −*i*/2 ∑_α_ Γ_α_, and Δ∑_C_ is the Coulomb correction term.^34^ We focus on the elastic cotunneling regime where Δ∑_C_ ≈ 0, making inelastic contributions negligible. Variations in conductance (*G*) and thermopower (*S*) stem directly from the poles and resonances of .

The molecular Green’s function, , is determined by exactly diagonalizing the molecular Hamiltonian projected onto relevant atomic orbitals:^34^
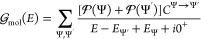
6where *E*_Ψ_ is the eigenvalue of the many-body molecular Hamiltonian *H*_mol_,  is the occupation probability of state Ψ, and *C*^Ψ→Ψ′^ represents the many-body transition matrix:
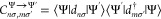
7where *d*_*nσ*_ annihilates an electron of spin σ on the *n*th atomic orbital. Here, Ψ and Ψ′ are eigenstates for *N* and *N* + 1 particle systems, respectively, with  given by the grand canonical ensemble in linear response.

The molecular Hamiltonian for the π-system was derived from first-principles using a renormalization procedure that incorporates off-resonant degrees of freedom (e.g., from the σ-system, image charge effects, etc.) implicitly as renormalized energy and coupling terms.^35^ In a basis of localized orbitals, the Hamiltonian can be written^35^
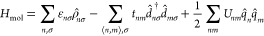
8where ε_*nσ*_ is the effective on-site potential for σ-spin electrons on orbital *n*, ρ̂_*nσ*_ = *d̂*_*nσ*_^†^*d̂*_*nσ*_, *ĝ*_*n*_ = (∑_σ_ ρ̂_*nσ*_) – 1 is the net charge operator, and *t*_*nm*_ are the effective tight-binding matrix elements. The Coulomb interaction *U*_*nm*_ between electrons in orbitals *n* and *m* is calculated via a multipole expansion, incorporating electrode image charge effects.^35^ These interactions are screened by a uniform dielectric ϵ, arising from the σ-electrons, with the screening surface set one covalent radius^36^ beyond the outermost gold nucleus.^37^

We consider transport experiments involving thousands of similarly prepared junctions, where bonding geometry, electromagnetic environment, chemical potentials, and molecular structure vary across the ensemble. When thermopower and conductance are measured simultaneously, the joint *GS* probability density function (PDF) can be constructed by binning the data^38,39^
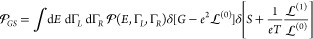
9where  represents the probability of realizing a junction with couplings Γ_*L*_, Γ_*R*_, and alignment energy *E*. We treat Γ_α_ and *E* as independent Gaussian random variables, so that , with each distribution characterized by its mean and standard deviation: ⟨Γ_α_⟩, σ_*Γα*_ and ⟨*E*⟩, σ_*E*_, respectively.

Beyond the marginal distributions of , we also examine correlations between simultaneously measured quantities. Pearson’s correlation coefficient measures the linear relationship between two variables *X* and *Y*, defined as
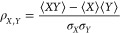
10where  is the standard deviation of *X*.

The conductance of a node possessing system is expected to decrease as the lead chemical potential *E* is tuned away from any resonances, such as the HOMO or LUMO resonance at *E*_H_ and *E*_L_, respectively, toward the particle-hole symmetric energy. In contrast, the magnitude of the thermopower |*S* |is predicted to initially decrease before reaching a peak value due to QI.^1^ For a quadratic node, this occurs when *E* – *E*_node_ ∼ ±*πkT*/√3, where *E*_node_ is the node energy.^2^ On the other hand, in systems without a node, |*S*| is expected to decrease monotonically, eventually reaching zero at the particle-hole symmetric energy. Therefore, in the node-possessing junction when *E* is closer to *E*_node_ than to *E*_L,*H*_, the inflection point in the thermopower spectrum should cause *G* and |*S*| to become strongly anticorrelated, whereas in junctions without a node they should be correlated. This suggests that the presence of a node can be identified by the condition
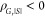
11

To investigate this method, we first compare two benzenediamine (BDA, C_6_H_8_N_2_) junctions coupled to Au electrodes in either the para (1,4-BDA) or meta (1,3-BDA) configurations. The 1,4-BDA junction, which is not expected to exhibit a midgap node, has been extensively studied both theoretically^40−42^ and experimentally.^32,43−46^ In contrast, the 1,3-BDA junction is predicted to have a node in the π-system’s transmission function due to QI effects.^5,7,14,34,47^ While the conductance of 1,3-BDA has been measured,^27^ its thermoelectric response has not.

The calculated room-temperature transmission , conductance *G*, and thermopower *S* of 1,4-BDA and 1,3-BDA junctions are shown as a function of electrode energy in Figure 1. Energy values are vacuum-referenced, and the calculations include all intermolecular many-body correlations exactly. Image charge effects reduce the gap in both junctions, breaking particle-hole symmetry due to the formation of N–Au dipoles. For 1,4-BDA, the gas-phase gap of 9.30 eV is reduced by 1.19 eV, shifting the HOMO *E*_H_ from −6.06 eV to −8.39 eV, consistent with experimental ionization potentials^48^ and other many-body calculations.^41,42,49^ In 1,3-BDA, the gap reduces by 0.66 eV, with *E*_H_ at −8.64 eV, and a node at *E*_node_ = −5.49 eV. Additional details regarding the computational methods are provided in the Supporting Information.

**Figure 1:**
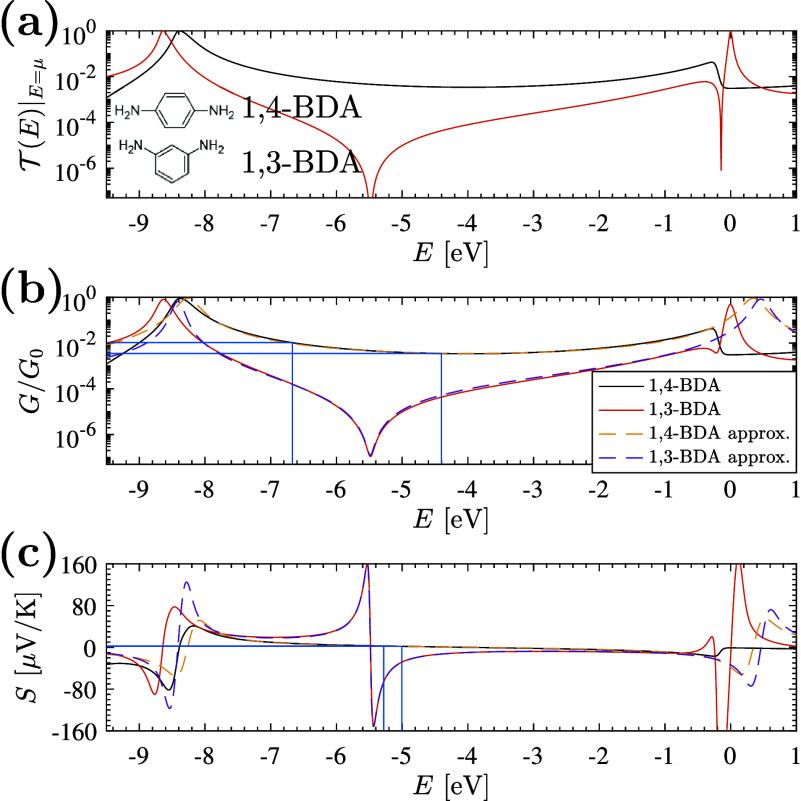
(a) Transmission function for para (1,4-BDA) and meta (1,3-BDA) junctions shown as a function of electrode chemical potential *E*, calculated using many-body MDE theory. Owing to quantum interference, the meta junction exhibits a quadratic node around *E*_node_ ∼ −5.49 eV. (b and c) Conductance (*G*) and thermopower (*S*) calculated using eq 1, with dashed lines representing the reduced-orbital model’s results. Horizontal lines correspond to experimentally observed values and vertical lines to their associated chemical potentials. In the para junction, *G* and |*S*| are correlated while in the meta junction they are anticorrelated when *E* is closer to the node than a molecular resonance. Simulations are conducted for junctions at 300 K, and energy values are relative to vacuum.

Both the conductance and thermopower of the 1,4-BDA junction have been measured, with ⟨*G*_para_^exp^ ⟩ = (7.0 ± 3.5) × 10^–3^*G*_0_^27,32,42−45^ and ⟨*S*_para_^exp^⟩ = 2.3 ± 0.3 μ V/*K*,^32,46,50^ as indicated by the solid blue lines. The small magnitude of the observed thermopower suggests that *E*_*F*_ is close to the particle-hole symmetric point and detuned from any molecular resonances, where *S* is nearly independent of Γ_α_.^1,3^ From the range of reported thermopower values, our calculated spectrum indicates *E* ∈[−5.28, – 5.005]eV, while fitting *G* to our calculations yields an even broader range of electrode chemical potentials, *E* ∈[−6.67, – 4.40]eV.

Given the computational challenges of calculating full many-body spectra for large ensembles of junctions, we focus on the low-energy region between HOMO and LUMO resonances to determine the statistical properties of the ensemble. In this regime, frontier orbitals dominate the response, allowing the molecular Green’s function to be approximated as^19^
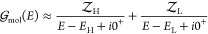
12where *E*_H,*L*_ are the HOMO and LUMO energies, and  are effective many-body factors (see Supporting Information). This simplifies eqs 3 and 5 to  and , respectively, where Γ̃_α_ are the effective lead couplings. Applying this approximation to the BDA junctions (dashed lines in Figure 1), we find excellent agreement with the many-body spectra over the low-energy region of interest.

Using this model, we simulated an ensemble of junctions to determine the statistical parameters that best match experimental observations. The calculated *G* and *S* histograms for 10,000 1,4-BDA and 1,3-BDA junctions are shown in Figure 2a. For the para junction, we find ⟨Γ̃_α_⟩ = 0.8 eV, σ_Γ̃α_ = 0.265 eV, ⟨*E*⟩ = −5.38 eV, and σ_*E*_ = 0.15 eV, yielding ⟨*G*_para_⟩ = (7.2 ± 3.4) × 10^–3^*G*_0_ and ⟨*S*_para_⟩ = 2.302 ± 0.299 μ V/K, closely matching experiment.^32,43−46^ For the meta junction, we predict ⟨*G*_meta_⟩ = (3.36 ± 4.7) × 10^–6^*G*_0_ and ⟨*S*_meta_⟩ = −36.8 ± 94 μ V/K, using para-junction parameters due to limited experimental data.^27^ The average lead chemical potential ⟨*E*⟩ aligns with the Au bulk work function (−5.3 eV), with σ_*E*_ close to Au crystal plane variation (0.16 eV),^51^ suggesting limited energetically favorable amine-Au bonding configurations.^27,44,50^

**Figure 2:**
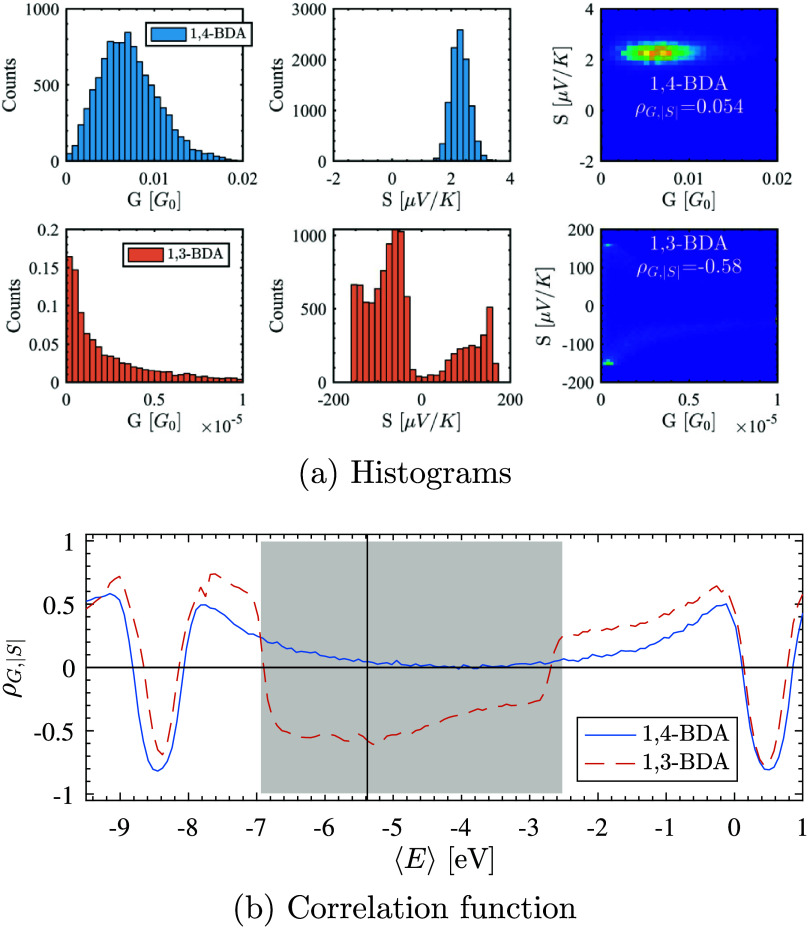
(a) Calculated conductance *G* and thermopower *S* distributions for 1,4-BDA and 1,3-BDA from an ensemble of 10,000 junctions. The reduced-orbital model, using parameters extracted from MDE many-body calculations, shows excellent agreement with experimental data.^32,43,44^ The 2D histogram of simultaneously measured *G* and *S* values yields correlation functions of 0.05 for the para junction and −0.58 for the meta junction. (b) The correlation function ρ_*G*,|*S*|_ as a function of average lead chemical potential ⟨*E* ⟩, with the Fermi energy of Au indicated by the vertical black line. The gray region, determined by eq 13, represents the range of validity expected for this method without statistical noise. Ensemble parameters are ⟨Γ̃_α_ ⟩ = 0.8 eV, σ_Γ̃_α__ = 0.265 eV, and σ_*E*_ = 0.15 eV. Junctions are not symmetrically coupled, and all calculations are performed at room temperature (*T* = 300 K).

Although conductance ratios and the observation of lower ⟨*G*⟩ with higher ⟨*S*⟩ in nodal versus anodal junctions have been used to indirectly identify transmission nodes,^21,23,52^ similar trends can arise from other factors, such as variations in lead-molecule coupling or level alignment distributions. Identifying QI from these trends is particularly challenging in systems with complex anchor groups,^52^ where coupling effects can differently influence nodal and anodal-junction transport. Likewise, in junctions with multiple accessible configurations^53^ or conformations,^54^ structural variations can obscure interference effects, again complicating the identification of transmission nodes. In contrast, analyzing each junction’s joint probability distribution  captures variations within the same ensemble, enabling unambiguous node identification through the sign of ρ_*G*, |*S*|_ across a wide energy range. For the BDA junctions shown in Figure 2a, we find correlation values of +0.05 for the para and −0.58 for the meta junction at the Fermi energy, clearly identifying the node in the meta junction. This correlation measure is not relative; nodes can be identified from a single junction’s distribution. This extends the method’s applicability to complex systems,^25,53,54^ potentially enabling investigations of metastable state formation or QI effects during single-molecule reactions.

While the presence of a node can often be understood in terms of molecular symmetry,^5,6,13,14,16,25,55−57^ this is not always the case.^15,17,18^ In general, both the energy and even the existence of a node depend strongly on how quantum many-body correlations are incorporated. Ideally, therefore, any effective method for identifying a node should be independent of the specific electronic structure approximations used and should not rely on the precise energy of the node.

Inflection points of the thermopower arise from resonances and nodes, which in turn stem from the mathematical structure of the exact Green’s function. Anticorrelations between *G* and |*S*| in the midgap region can therefore serve as a direct indication of a node so long as |*E* – *E*_node_| is bounded by the inflection point energies of *S*. Specifically, in an anodal junction, ρ_*G*, |*S*|_ remains positive for all energies, whereas in a nodal junction, ρ_*G*,|*S*|_ becomes negative, provided
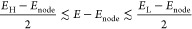
13where the condition |*E* – *E*_node_| > *π kT*/√3 is omitted since σ_*E*_ ≫ *kT* at room temperature, and we approximate the inflection point energy as the midpoint between the nodal and molecular resonance levels. Figure 2b shows the correlation function as a function of ⟨*E*⟩, with ρ_*G*,|*S*|_ < 0 for the meta junction over a broad range of lead chemical potentials.

Anticorrelations between *G* and |*S* |identify a node only within this specific energy range. As shown, ρ_*G*,|*S*|_ < 0 can also occur near a resonance, meaning the method may fail if the electrodes Fermi energy is closer to a resonance than the node. To illustrate, we analyzed para- and meta-configured Au-benzenedithiol-Au junctions,^44,58−61^ where |*E* – *E*_node_| is too large to observe ρ_*G*,|*S*|_ < 0 (see Supporting Information).

As we have demonstrated, anticorrelations between simultaneously measured *G* and *S* values are an effective indicator of the presence of a node, even for nodes far detuned from the chemical potential of the electrodes. However, within the energy range above, there are two ways this method can fail: (1) in a junction without a node, ρ_*G*,|*S*|_ < 0; and (2) in a junction with a node, ρ_*G*,|*S*|_ > 0. The first case occurs when variations in *G* or *S* are small when compared to the standard error of ρ_*G*,|*S*|_, which scales as *N*^–1/2^ with *N* being the number of measurements. Typically, this occurs when fluctuations over the ensemble are predominantly from variations in Γ_α_, i.e. when σ_*Γα*_ > σ_*E*_. Since, away from resonance, *S* is nearly independent of Γ_α_, ρ_*G*,|*S*|_ ∼ 0, which can erroneously become a small negative value due to statistical noise in an anodal junction. The second case occurs when the node-possessing channels transport is subsumed by other processes, e.g. from additional molecular orbitals, incoherent transport, direct electrode-electrode, H-bond, or through-space tunneling, which effectively mask the transmission node. In this scenario, the total transmission can be expressed as
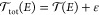
14where  represents the transmission of the node-possessing channel, and ε is the additional transport term. When  over the region of interest, the nodes signature is effectively washed out.

The influence of σ_*E*_, σ_*Γα*_ and ε on ρ_*G*,|*S*|_ for ensembles of meta and para configured BDA junctions are shown as a function of average lead chemical potential ⟨*E*⟩ in the three panels of Figure 3, respectively. As indicated in the bottom panels of Figure 3 (a) and (b), when variations in *S* are small, statistical errors can dominate leading to ρ_*G*,|*S*|_≲0 in the 1,4-BDA junction. The influence of an additional transmission channel ε is shown in Figure 3c. The correlation function is sensitive to relative changes rather than absolute variations; therefore, as expected from the transmission function, variations in the conductance stemming from the node are obscured when ε ≳10^–5^. In that case, both the para and meta junctions exhibit positive correlations over the midgap energy range.

**Figure 3:**
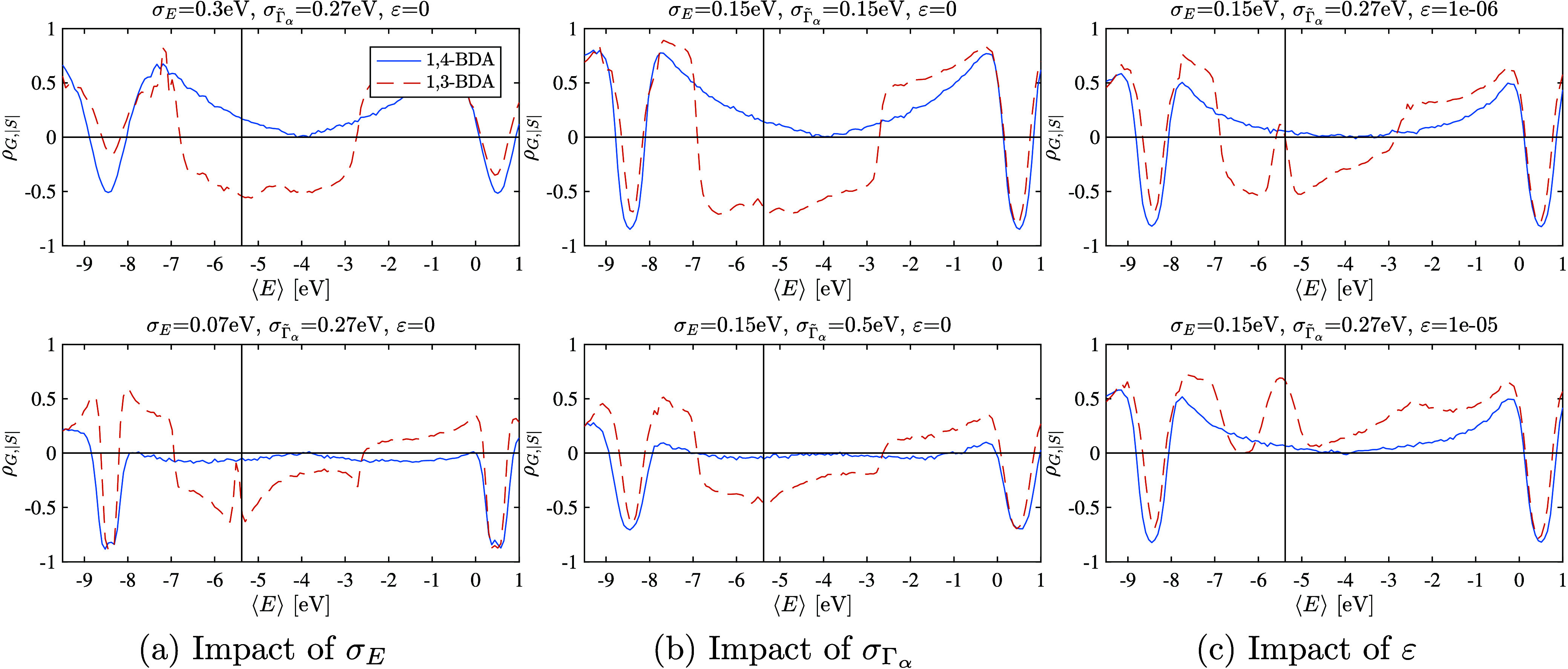
Effect of σ_*E*_, σ_Γ̃_α__, and the additional channel’s transmission probability ε on the correlation coefficient ρ_*G*,|*S*|_ for 1,4-BDA and 1,3-BDA junctions. (a) As σ_*E*_ decreases below σ_Γ̃_α__, the variations in *S* become sufficiently small that statistical noise may cause ρ_*G*,|*S*|_ ≲ 0 even in an anodal junction. (b) A similar trend is observed as σ_*Γα*_ increases while σ_*E*_ remains fixed, where false positives are possible for ρ_*G*,|*S*|_ ∼ 0 due to statistical uncertainties. (c) The introduction of an additional (energy-independent) channel with transmission probability ε tends to wash out the nodal signature. When ε is large relative to the node-induced transmission (here, ε ≳ 10^–5^) the nodal signal is obscured and ρ_*G*,|*S*|_ > 0 for both junctions. All results are based on ensembles of 10,000 simulated junctions using the reduced-orbital model with ⟨Γ̃_α_⟩ = 0.8 eV. Junctions were not assumed to be symmetrically coupled, and calculations are for systems operating at 300 K.

Although our node-identification method is broadly applicable, it requires simultaneously measuring *G* and *S* from a node-possessing transport channel.^24,25^ This may be feasible in BDA-based junctions, but our parameters suggest that additional mechanisms, such as direct electrode-electrode or through-space tunneling, may also play a significant role when the Fermi energy is near a node. It is also possible that the  from vacuum tunneling^62^ can be distinguished from the node-possessing channel, allowing direct tunneling data to be filtered out, with rational chemical design further reducing these effects. However, since these effects also decrease exponentially with molecular length, larger systems should enable direct observation of interference effects.^24,25^ Therefore, SMJs based on larger molecules may be preferable, particularly when working with small ensembles.

As a second example, we examine the para- and meta-configured 4,4′-diiodo-*p*-terphenyl and 4,4′-diiodo-*m*-terphenyl molecules (Figure 4a). Although iodine may complicate transport measurements,^63^ we include these junctions due to observed nodes in these systems.^25^ Conductance histograms from 1,000 junctions, calculated using our reduced-orbital model, are shown in Figure 4b. Resonance energies *E*_H,*L*_ and node energy *E*_node_ were extracted from measurements and DFT+Σ calculations reported in ref (25). For the para junction, we find ⟨*G*_para_⟩ = (2.79 ± 0.195) × 10^–4^*G*_0_ with ensemble parameters ⟨Γ̃_α_⟩ = 34.5 meV, σ_Γ̃_α__ = 1.6 meV, and σ_*E*_ = 0.15 eV. For the meta junction, ⟨*G*_meta_⟩ = (8.6 ± 0.25) × 10^–5^*G*_0_ with ⟨Γ̃_α_⟩ = 96 meV, σ_Γ̃_α__ = 1 meV, and σ_*E*_ = 7 meV, matching experimental data.^25^ Using these parameters, we also find ⟨*S*_para_⟩ = 1.357 ± 1.06 μV/K and ⟨*S*_meta_⟩ = −26.14 ± 0.33 μV/K.

**Figure 4:**
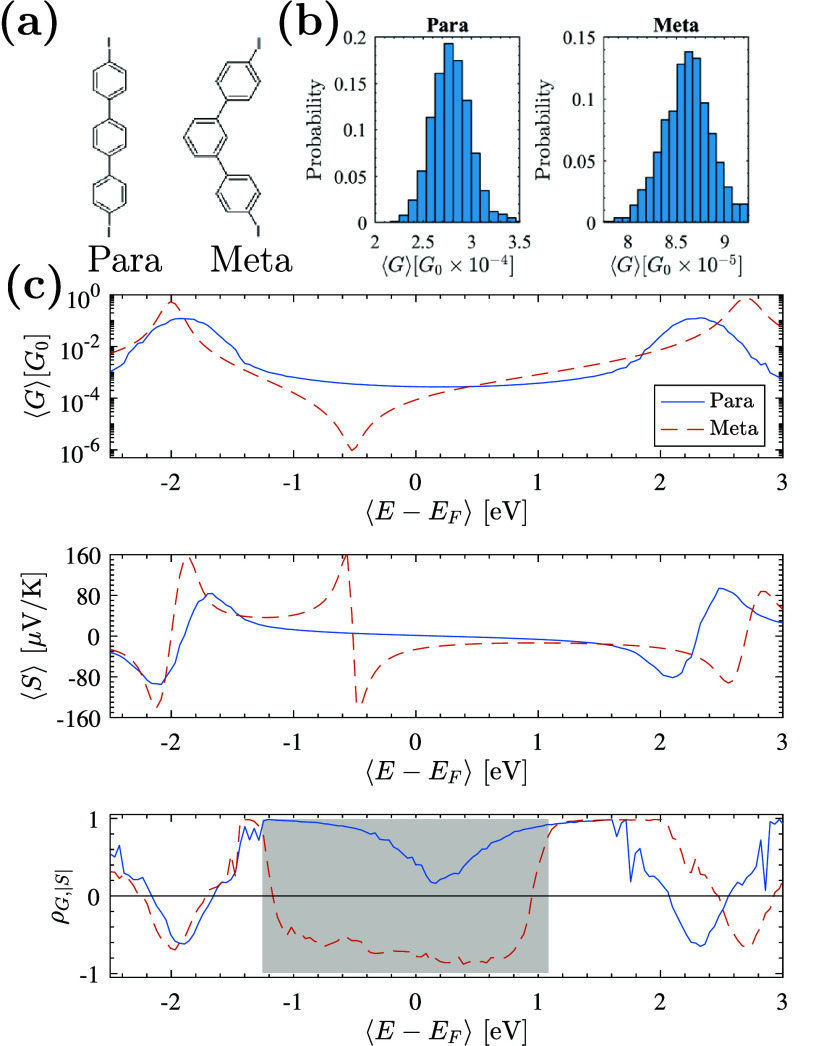
(a) Schematic diagrams of the para (4,4′-diiodo-*p*-terphenyl) and meta (4,4′-diiodo-*m*-terphenyl) junctions. In both the meta and para junctions we consider molecules with a 180° dihedral angle for the middle phenyl ring. (b) Conductance histograms for 1,000 junction ensembles with ⟨*G*_para_⟩ = (2.79 ± 0.21) × 10^–4^*G*_0_ and ⟨*G*_meta_⟩ = (8.6 ± 0.25) × 10^–5^*G*_0_, in excellent agreement with experiment.^25^ (c) The average conductance ⟨*G*⟩, thermopower ⟨*S*⟩, and correlation function ρ_*G*,|*S*|_, where the nodal condition (ρ_*G*,|*S*|_ < 0) unambiguously identifies the nodes over a wide spectral range and from meta-junction measurements alone. The region in gray is where we expect ρ_*G*,|*S*|_ < 0 in ensembles without noise. Junctions are not assumed to be symmetrically coupled, and calculations are for systems operating at 300 K.

Direct electrode-electrode tunneling and off-resonant transport, such as through the σ-channel, decrease exponentially with molecular length, enabling direct observation of the node in the meta junction via changes in ⟨*G*⟩ with the local gate potential.^25^ However, even without gating, we predict a clear nodal signature from meta data alone, with ρ_*G*,|*S*|_^para^ = 0.398 and ρ_*G*,|*S*|_^meta^ = −0.727 at the Fermi energy. Figure 4c illustrates ⟨*G*⟩, ⟨*S*⟩, and ρ_*G*,|*S*|_ as functions of lead chemical potential, highlighting the node in the meta system where ρ_*G*,|*S*|_ < 0 across a broad energy range. This anticorrelation signature of a transmission node is both reliable and versatile, with similar outcomes expected in other systems.^23,24^

In conclusion, we have demonstrated that anticorrelations between simultaneously measured conductance (*G*) and thermopower (*S*) provide a robust and generally applicable method to unambiguously identify transmission nodes in molecular junctions. Utilizing many-body theory and a reduced-orbital model to capture low-energy transport, we calculate transport distributions for para- and meta-configured benzenediamine junctions and larger diiod*o*-terphenyl systems. We find the condition ρ_*G*,|*S*|_ < 0 reliably indicates destructive QI, even in the presence of significant uncertainties in energy alignment, lead-molecule coupling, and chemical configuration.

We also explored the limitations of this metric, noting that while anticorrelations between *G* and |*S*| occur near nodes or resonances in accordance with the exact transport equations, the statistical method may fail if noise exceeds variations in *G* or *S* within the ensemble. Additionally, the methods efficacy is reduced if transport is dominated by anodal contributions, such as incoherent tunneling, direct electrode-electrode tunneling, or through-space tunneling. In the former case, small but negative ρ_*G*,|*S*|_ values may appear in anodal junctions with limited ensemble sizes, while in the latter, positive ρ_*G*,|*S*|_ values can occur even in junctions with a node.

Despite these limitations, this method enables the identification of nodes from a single joint ensemble, eliminating the need for isomer comparisons. The nodal signature is robust and directly tied to transport theory, facilitating direct comparisons between experimental data and theoretical predictions. Additionally, since interference affects *G* and *S* differently across a wide energy range, this method is broadband, enabling the identification of far-detuned nodes. This capability allows QI features to be unambiguously identified and investigated, even in systems where a precise determination of the nodal energy may be challenging or computationally infeasible to determine.

## Supporting Information Available

Additional details regarding the derivation of the molecular Hamiltonian, the reduced-orbital model, and an analysis of para- and meta-configured benzenedithiol (BDT) junctions (PDF)
