# Supplementary material for: Identifying Quantum Interference Effects from Joint Conductance–Thermopower Statistics
Source: Nano Lett. 2024 Nov 13;24(47):15110–7. doi: 10.1021/acs.nanolett.4c04439 (PMC11613687; doi:10.1021/acs.nanolett.4c04439)
Supplement: Supplementary file 1 — nl4c04439_si_001.pdf [file nl4c04439_si_001.pdf]

Identifying Quantum Interference Effects from Joint Conductance-Thermopower Statistics

Justin P. Bergfield*,†,‡

†*Department of Physics, Illinois State University, Moulton Hall, Normal, IL, 61761, USA*

‡*Department of Chemistry, Illinois State University, Julian Hall, Normal, IL, 61761, USA*

E-mail: jpbergf@ilstu.edu

Molecular Hamiltonian

The Hamiltonian for the benzenedithiol molecules considered in this study were derived from *first principles* using the effective field theory for the π-system (π-EFT) as developed in Ref. 1. Here, we provide a brief overview. Under the Born-Oppenheimer approximation, the electronic Hamiltonian for an isolated molecule can be expressed as:

$$H_{\rm mol} = H^{(1)} + H^{(2)}, \tag{1}$$

where the one-body term is given by:

$$H^{(1)} = \sum_{nm\sigma} H_{nm\sigma} \hat{d}^{\dagger}_{n\sigma} \hat{d}_{m\sigma} \tag{2}$$

with

$$H_{nm\sigma} = \sum_{kl} S^{-1}_{nk\sigma} H^{(1)}_{kl\sigma} (S^{-1}_{ml\sigma})^* \tag{3}$$

and

$$H^{(1)}_{nm\sigma} = \int d^3 r \phi^*_{n\sigma}(\vec{r}) \left(-\frac{\hbar^2}{2m} \nabla^2 + V \right) \phi_{m\sigma}(\vec{r}). \tag{4}$$

Here, $S_{nm} = \langle \phi_n | \phi_m \rangle$ represents the overlap matrix element between atomic orbitals $\phi_{n,m}$. In the main text, we simplify by setting $\varepsilon_{n\sigma} = H^{(1)}_{nn\sigma}$ and $t_{nm} = \sum_\sigma H^{(1)}_{nm\sigma}$.

The two-body term is represented in the atomic orbital basis as:

$$H^{(2)} = \frac{1}{2} \sum_{nmlk\sigma\sigma'} U_{nmlk} \hat{d}^\dagger_{n\sigma} \hat{d}^\dagger_{m\sigma'} \hat{d}_{l\sigma'} \hat{d}_{k\sigma}, \tag{5}$$

where

$$U_{nmkl} = \int d^3 r_1 d^3 r_2 \phi^*_n(\vec{r}_1) \phi^*_m(\vec{r}_2) \times \frac{e^2}{|r_1 - r_2|} \phi_k(\vec{r}_2) \phi_l(\vec{r}_1) \tag{6}$$

and

$$U_{nmkl} = \sum_{opqr} S^{-1}_{no} S^{-1}_{mp} U_{opqr} (S^{-1}_{kq})^* (S^{-1}_{lr})^* \tag{7}$$

The effective Hamiltonian for the π-electron system is derived by excluding non-bonding orbitals and expanding the interaction matrix in terms of the interatomic bond length. This reduces the interaction matrix to $U_{nm} = \delta_{nl} \delta_{mk} U_{nmkl}$ through the renormalization of atomic orbitals.[1] This method is mathematically equivalent to the neglect of differential overlap approximation,[2–4] but π-EFT only requires the effective Hamiltonian to be local.[1]

Expanding the interaction matrix in terms of bond length gives the multipole expansion:

$$
\begin{aligned}
U_{nm} &= U_{nn}\delta_{nm} \\
&+ (1-\delta_{nm})\left(U_{nm}^{MM} + U_{nm}^{QM} + U_{mn}^{QM} + U_{nm}^{QQ}\right) \\
&+ \mathcal{O}(r^{-6}),
\end{aligned} \tag{8}
$$

where U_{nm}^{MM} is the monopole-monopole interaction, U_{nm}^{QM} the quadrupole-monopole interaction, and U_{nm}^{QQ} the quadrupole-quadrupole interaction between renormalized π-orbitals, as detailed in Ref. 1. Imposing particle-hole symmetry on the isolated molecule, justified by the success of other semi-empirical methods (e.g., Pariser-Parr-Pople (PPP)-type[5,6]), and adding a Zeeman term, yields the effective molecular Hamiltonian used in the main text.

Although this Hamiltonian is formally similar to certain semi-empirical methods (e.g. the Pariser–Parr–Pople or "extended Hubbard" methods),[6–8] this is an *ab initio* theory which accurately describes arbitrary π-electron systems.[1] Moreover, the π-electron quadrupole moment Q is a physical quantity which takes the place of the *ad hoc* interaction parameterizations used in other models.

The π-EFT parameters were determined via a renormalization procedure fitting experimental data to quantities accurately represented by a π-electron-only model. Specifically, we optimized predictions for 1) the vertical ionization energy, 2) the vertical electron affinity, and 3) the six lowest singlet and triplet excitations of the neutral gas-phase benzene molecule.[1] This fit, comparable or superior to traditional PPP models,[9] yields $U_{nn} = 9.69$ eV for on-site repulsion, $t = 2.70$ eV for nearest-neighbor hopping, $\varepsilon = 1.56$ eV for the dielectric constant, and $Q = -0.65e\text{Å}^2$ for the π-electron quadrupole moment. These values, consistent with previous π-electron models,[5,9] and the new parameter Q governing short-distance interaction corrections, were used for both molecules in this study.

Electrostatically, the Au electrodes are modeled as metallic spheres with radii of 0.5 nm.

The partially ionic character of the Au-N bond was accounted for by placing point charges of $-0.85e$ at the nitrogen atoms. This value was determined via simultaneous fit of the fundamental gap of benzenediamine (BDA) in gas-phase in the junction (both with and without screening within the electrodes)[10–12] and the experimentally determined thermopower and conductance[13–17] values. Note that this value is consistent with previous calculations[1] of benzenedithiol, since nitrogen tends to form more polar bonds than sulfur and is more electronegative.[1] Using this method the trace of each electrode's tunnel coupling matrix is set to 0.6eV. This value of $\mathrm{Tr}\{\Gamma_\alpha\}$ is larger than that found using the same method for benzeneditihiol (0.44eV), a surprise given the binding energy of Au-S is expected to by ∼5 times that of Au-N.[18] Although the results of this work do not depend on this value in any critical way, this may be a consequence of taking the wide-band limit wherein Γ_α is assumed to be independent of energy. It may also be a reflection of the reduced binding configurations of amine as compared with thiol. The same tunnel-coupling strength was used for both the 1,4-BDA and 1,3-BDA junctions.

Molecular geometries were based on published density functional theory (DFT) calculation results. For the 1,4-BDA junction, the C-C bond length of 1.41 Åwas used as reported in previous studies,[19] along with a C-N bond length of 1.43 Å.[19–21] The Au-N bond length was fixed to 2.43 Å.[21,22] For the 1,3-BDA junction, the Au-N bond length was kept consistent with the 1,4-BDA junction, with the only difference being the change in the binding site.

Reduced-orbital model

We focus on room-temperature transport through SMJs composed of small organic molecules whose primary conducting channel has a node far detuned from any molecular addition or removal energy. In this regime, the low-energy transport is dominated by the HOMO and LUMO resonances, whose tails gives rise to a quadratic node in that channel.[23–25,25–28] Here,

we develop two equivalent techniques to describe the low-energy transport.

Method 1: Effective Molecular Hamiltonian and Green's Function

The first method involves constructing an effective "molecular Hamiltonian for the system, represented as:

$$\mathcal{H}_{\text{mol}} = \begin{pmatrix} a & -t \\ -t & b \end{pmatrix}, \tag{9}$$

where a and b are the onsite energies of the molecular orbitals, and t is the hopping parameter between them. The coupling to the electrodes is captured by the Γ matrix, which only connects to the first orbital:

$$\mathbf{\Gamma} = \begin{pmatrix} \Gamma_{\text{tot}} & 0 \\ 0 & 0 \end{pmatrix}. \tag{10}$$

where $\Gamma_{\text{tot}} = \Gamma_{\text{L}} + \Gamma_{\text{R}}$. The full Green's function of the system is then calculated using the Dyson equation:

$$\mathcal{G}(E) = \left[E\mathbb{I} - \mathcal{H}_{\text{mol}} + \frac{i}{2}\mathbf{\Gamma} \right]^{-1}, \tag{11}$$

where $\mathbb{I}$ is the identity matrix. The transmission function, $\mathcal{T}(E)$ is then found via

$$\mathcal{T}(\mu) = \Gamma_L \Gamma_R \left| \mathcal{G}_{11}(\mu) \right|^2. \tag{12}$$

In the meta-configured junction, the energy position of the node can be controlled by the parameter b, and the hopping parameter t is determined by ensuring the correct HOMO-LUMO gap, which is crucial for accurate modeling of the low-energy transport properties. Specifically, the values of a and t that produce the correct HOMO and LUMO energies are

given by:

$$b = \mu_0 \tag{13}$$

$$a = -b + E_{\mathrm{H}} + E_{\mathrm{L}}, \tag{14}$$

$$t = \sqrt{(b - E_{\mathrm{H}})(E_{\mathrm{L}} - b)}, \tag{15}$$

where μ_0 is the node energy, and E_{H} and E_{L} are the energies of the HOMO and LUMO levels, respectively.

For the para-configured junction,

$$a = \frac{E_{\mathrm{H}} + E_{\mathrm{L}}}{2}, \tag{16}$$

$$t = \frac{1}{2}\sqrt{(E_L - E_H)^2 + \left(\frac{\Gamma_{\mathrm{tot}}}{2}\right)^2}. \tag{17}$$

This approach allows us to explicitly set the node's energy via the parameter b, providing a flexible and precise method to explore the effects of the quadratic node on the system's transport properties.

Method 2: Approximate Molecular Green's Function

This method is derived in detail in Ref. 24. In this approximation, the molecular Green's function is written as sum of contributions from the HOMO and LUMO states:

$$\mathcal{G}_{\mathrm{mol}}(E) = \frac{\mathcal{Z}_H}{E - E_H + i0^+} + \frac{\mathcal{Z}_L}{E - E_L + i0^+}, \tag{18}$$

where $\mathcal{Z}_{H,L}$ are the effective manybody factors. With this molecular Green's function transmission is then:

$$\mathcal{T}(E) = \frac{4\Gamma_L\Gamma_R\left(B\cdot E_{\mathrm{H}} + A\cdot E_{\mathrm{L}} - E\right)^2}{4\cdot(E_{\mathrm{H}}-\mu)^2\cdot(E_{\mathrm{L}}-\mu)^2 + \left(B\cdot E_{\mathrm{H}} + A\cdot E_{\mathrm{L}} - \mu\right)^2\cdot\Gamma_{\mathrm{tot}}^2} \tag{19}$$

From this expression we see that in the limit of a single resonance (i.e $E_{\mathrm{L}} \to \infty$), the full width at half maximum is $A\Gamma_{\mathrm{tot}}$, suggesting that $A = 1$. Solving for the case that $\mathcal{T} = 0$ gives:

$$\frac{B}{A} = -\frac{E_{\mathrm{L}} - E_{\mathrm{node}}}{E_{\mathrm{H}} - E_{\mathrm{node}}}, \tag{20}$$

where E_{node} is the nodal energy. In the anodal junction $A = -B$. Plugging these in gives

$$\mathcal{T}_{\mathrm{para}}(E) = \frac{4\Gamma_L\Gamma_R U^2}{4(E_{\mathrm{H}}-E)^2(E-E_{\mathrm{L}})^2 + \left(\frac{\Gamma_{\mathrm{tot}}}{2}\right)^2 U^2} \tag{21}$$

$$\mathcal{T}_{\mathrm{meta}}(E) = \frac{\Gamma_L\Gamma_R U^2(E-E_{\mathrm{node}})^2}{(E-E_{\mathrm{H}})^2(E_{\mathrm{H}}-E_{\mathrm{node}})^2(E-E_{\mathrm{L}})^2 + \left(\frac{\Gamma_{\mathrm{tot}}}{2}\right)^2 U^2(E-E_{\mathrm{node}})^2}, \tag{22}$$

where $U = E_{\mathrm{L}} - E_{\mathrm{H}}$

Para- and Meta-configured benzenedithiol (BDT)

We examine the response of para- and meta-configured benzenedithiol (BDT) junctions, focusing on the experimentally measured conductance and thermopower of the para-configured 1,4-BDT. Reported values are $G_{\mathrm{para}} = 0.011 \pm 50\%\ G_0$ and $S_{\mathrm{para}} = 7 \pm 0.2\ \mu\mathrm{V/K}$.[29,30] Using our MDE many-body theory in conjunction with the π-EFT Hamiltonian discussed previously, we find excellent agreement with these experimental values, as well as with the experimentally observed ionization potential and electron affinity.[1]

We begin by aligning the gas-phase energy levels of BDT with the work function (WF) of the Au electrodes. This alignment is based on the difference between benzene's ionization

potential (~9.24 eV) and the average WF over Au crystal planes (~5.3 eV). We then adjust the mean and distribution of the chemical potential until the simulated thermopower matches the observed value. The resulting mean and standard deviation of the chemical potential distribution are $\langle\mu\rangle = -2.885$ eV and $\sigma_\mu = 0.16$ eV, respectively, with σ_μ assumed to be dominated by the WF variation of the Au crystal planes.[31] Using these values, our calculated thermopower is $\langle S\rangle = 7$ μV/K (FWHM $\sim$ 2.26 μV/K), which agrees with the measured data.[30]

By calibrating our model to these experimental results, we determine the average lead chemical potential to be $\langle E\rangle = -5.3$ eV, with a standard deviation $\sigma_\mu = 0.15$ eV, corresponding to the work function variation of the Au crystal planes.[31] The coupling strengths are estimated as $\langle\Gamma_\alpha\rangle = 0.44$ eV with $\sigma_{\Gamma_\alpha} = 0.2$ eV.

With the ensemble parameters we can explore the average response. The average conductance $\langle G\rangle$, thermopower $\langle S\rangle$, and correlation function $\rho_{G,|S|}$ are presented in Fig. 1 as a function of the average lead chemical potential $\langle E\rangle$. At the Fermi energy, indicated by the vertical black line, we find the following values for the para configuration:

$$\langle G_{\text{para}}\rangle = (4.45 \pm 2.95) \times 10^{-3} G_0, \tag{23}$$

$$\langle S_{\text{para}}\rangle = 6.983 \pm 0.5933\, \mu V/K \tag{24}$$

Applying the same parameters to the meta-configured junction yields:

$$\langle G_{\text{meta}}\rangle = (3.16 \pm 2.18) \times 10^{-4} G_0, \tag{25}$$

$$\langle S_{\text{meta}}\rangle = -11.51 \pm 0.2303\, \mu V/K \tag{26}$$

The Pearson correlation coefficients for the para and meta junctions at the Fermi energy are $\rho_{G,|S|} = 0.33$ and $\rho_{G,|S|} = 0.9801$, respectively. Notice that these values are both positive,

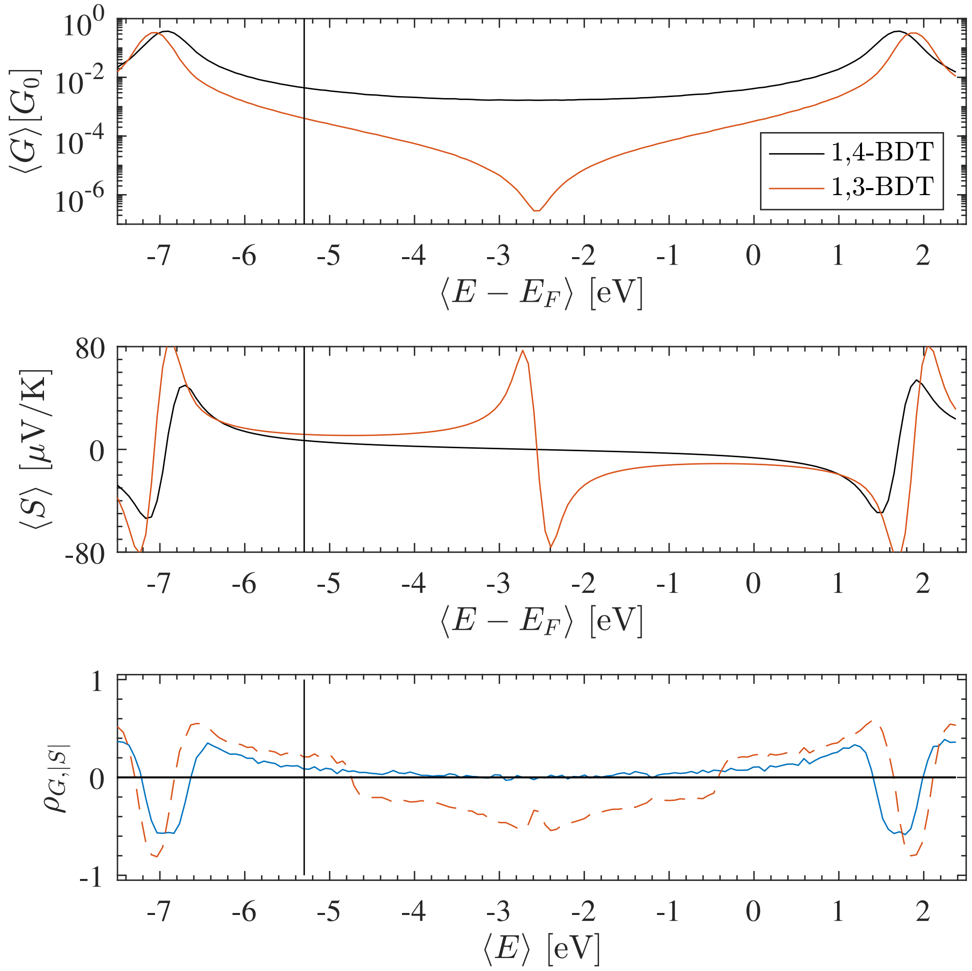

Figure 1: Manybody transport through BDT. Using Josh parameters[1]

which indicates that the energy is closer to the HOMO resonance than to the node, causing our method to miss the node in this particular case.

References

(1) Barr, J. D.; Stafford, C. A.; Bergfield, J. P. Effective field theory of interacting π electrons. *Phys. Rev. B* **2012**, *86*, 115403.

(2) Roby, K. On the justifiability of neglect of differential overlap molecular orbital methods. *Chem. Phys. Lett.* **1971**, *11*, 6–10.

(3) Pople, J. A.; Santry, D. P.; Segal, G. A. Approximate self-consistent molecular orbital theory. I. Invariant procedures. *J. Chem. Phys.* **1965**, *43*, S129–S135.

(4) Pople, J. A.; Segal, G. A. Approximate self-consistent molecular orbital theory. II. Calculations with complete neglect of differential overlap. *J. Chem. Phys.* **1965**, *43*, S136–S151.

(5) Ohno, K. Some remarks on the Pariser-Parr-Pople method. *Theor. Chim. Acta* **1964**, *2*, 219–227.

(6) Castleton, C.; Barford, W. Screening and the quantitative π-model description of the optical spectra and polarizations of phenyl based oligomers. *J. Chem. Phys.* **2002**, *117*, 3570–3582.

(7) Ramasesha, S.; Albert, I.; Sinha, B. Optical and magnetic properties of the exact PPP states of biphenyl. *Mol. Phys.* **1991**, *72*, 537–547.

(8) Bursill, R. J.; Castleton, C.; Barford, W. Optimal parametrisation of the Pariser–Parr–Pople Model for benzene and biphenyl. *Chem. Phys. Lett.* **1998**, *294*, 305–313.

(9) Barford, W. *Electronic and optical properties of conjugated polymers*; OUP Oxford, 2005; Vol. 129.

(10) Strange, M.; Rostgaard, C.; Häkkinen, H.; Thygesen, K. S. Self-consistent GW calculations of electronic transport in thiol-and amine-linked molecular junctions. *Phys. Rev. B* **2011**, *83*, 115108.

(11) Markussen, T.; Jin, C.; Thygesen, K. S. Quantitatively accurate calculations of conductance and thermopower of molecular junctions. *Phys. Status Solidi B Basic Res.* **2013**, *250*, 2394–2402.

(12) Pham, T. A.; Nguyen, H.-V.; Rocca, D.; Galli, G. GW calculations using the spectral decomposition of the dielectric matrix: Verification, validation, and comparison of methods. *Phys. Rev. B* **2013**, *87*, 155148.

(13) Kiguchi, M.; Miura, S.; Takahashi, T.; Hara, K.; Sawamura, M.; Murakoshi, K. Conductance of single 1, 4-benzenediamine molecule bridging between Au and Pt electrodes. *J. Phys. Chem. C* **2008**, *112*, 13349–13352.

(14) Venkataraman, L.; Klare, J. E.; Tam, I. W.; Nuckolls, C.; Hybertsen, M. S.; Steigerwald, M. L. Single-molecule circuits with well-defined molecular conductance. *Nano Lett.* **2006**, *6*, 458–462.

(15) Tsutsui, M.; Yokota, K.; Morikawa, T.; Taniguchi, M. Roles of vacuum tunnelling and contact mechanics in single-molecule thermopower. *Sci. Rep.* **2017**, *7*, 44276.

(16) Venkataraman, L.; Klare, J. E.; Nuckolls, C.; Hybertsen, M. S.; Steigerwald, M. L. Dependence of single-molecule junction conductance on molecular conformation. *Nature* **2006**, *442*, 904.

(17) Kiguchi, M.; Nakamura, H.; Takahashi, Y.; Takahashi, T.; Ohto, T. Effect of anchoring group position on formation and conductance of a single disubstituted benzene molecule bridging Au electrodes: change of conductive molecular orbital and electron pathway. *J. Phys. Chem. C* **2010**, *114*, 22254–22261.

(18) Chen, F.; Li, X.; Hihath, J.; Huang, Z.; Tao, N. Effect of anchoring groups on single-molecule conductance: comparative study of thiol-, amine-, and carboxylic-acid-terminated molecules. *J. Am. Chem. Soc.* **2006**, *128*, 15874–15881.

(19) Ning, J.; Li, R.; Shen, X.; Qian, Z.; Hou, S.; Rocha, A.; Sanvito, S. First-principles calculation on the zero-bias conductance of a gold/1, 4-diaminobenzene/gold molecular junction. *Nanotech.* **2007**, *18*, 345203.

(20) Colapietro, M.; Domenciano, A.; Portalone, G.; Schultz, G.; Hargittai, I. Molecular structure of p-diaminobenzene in the gaseous phase and in the crystal. *Journal of Physical Chemistry* **1987**, *91*, 1728–1737.

(21) Silva, C. E.; Pontes, R. B. Structural, electronic and transport properties of a single 1, 4-benzenediamine molecule attached to metal contacts of Au, Ag and Cu. *Computational Materials Science* **2020**, *171*, 109212.

(22) Quek, S. Y.; Venkataraman, L.; Choi, H. J.; Louie, S. G.; Hybertsen, M. S.; Neaton, J. Amine-gold linked single-molecule circuits: experiment and theory. *Nano Lett.* **2007**, *7*, 3477–3482.

(23) Pedersen, K. G.; Borges, A.; Hedegård, P.; Solomon, G. C.; Strange, M. Illusory connection between cross-conjugation and quantum interference. *J. Phys. Chem. C* **2015**, *119*, 26919–26924.

(24) Barr, J. D.; Stafford, C. A. On Transmission Node Structure in Interacting Systems. *arXiv preprint arXiv:1303.3618* **2013**, Accessed: 2024-10-20.

(25) Liu, J.; Huang, X.; Wang, F.; Hong, W. Quantum interference effects in charge transport through single-molecule junctions: detection, manipulation, and application. *Acc. Chem. Res.* **2018**, *52*, 151–160.

(26) Bergfield, J. P.; Solomon, G. C.; Stafford, C. A.; Ratner, M. A. Novel quantum interference effects in transport through molecular radicals. *Nano Lett.* **2011**, *11*, 2759–2764.

(27) Markussen, T.; Stadler, R.; Thygesen, K. S. The relation between structure and quantum interference in single molecule junctions. *Nano Lett.* **2010**, *10*, 4260–4265.

(28) Li, Y.; Buerkle, M.; Li, G.; Rostamian, A.; Wang, H.; Wang, Z.; Bowler, D. R.; Miyazaki, T.; Xiang, L.; Asai, Y.; others Gate controlling of quantum interference and

direct observation of anti-resonances in single molecule charge transport. *Nat. Mater.* **2019**, *18*, 357–363.

(29) Xiao, X.; Xu, B.; Tao, N. J. Measurement of single molecule conductance: Benzenedithiol and benzenedimethanethiol. *Nano Lett.* **2004**, *4*, 267–271.

(30) Baheti, K.; Malen, J. A.; Doak, P.; Reddy, P.; Jang, S.-Y.; Tilley, T. D.; Majumdar, A.; Segalman, R. A. Probing the chemistry of molecular heterojunctions using thermoelectricity. *Nano Lett.* **2008**, *8*, 715–719.

(31) Rankin, D. W. CRC handbook of chemistry and physics, edited by David R. Lide. 2009.
